# Uromodulin biology

**DOI:** 10.1093/ndt/gfae008

**Published:** 2024-01-11

**Authors:** Artemios G Karagiannidis, Marieta P Theodorakopoulou, Eva Pella, Pantelis A Sarafidis, Alberto Ortiz

**Affiliations:** First Department of Nephrology, Hippokration Hospital, Aristotle University of Thessaloniki, Thessaloniki, Greece; First Department of Nephrology, Hippokration Hospital, Aristotle University of Thessaloniki, Thessaloniki, Greece; First Department of Nephrology, Hippokration Hospital, Aristotle University of Thessaloniki, Thessaloniki, Greece; First Department of Nephrology, Hippokration Hospital, Aristotle University of Thessaloniki, Thessaloniki, Greece; Department of Nephrology and Hypertension, IIS-Fundacion Jimenez Diaz UAM, Madrid, Spain

**Keywords:** hypertension, roles, sodium sensitivity, Tamm–Horsfall protein, uromodulin

## Abstract

Uromodulin is a kidney-specific glycoprotein which is exclusively produced by the epithelial cells lining the thick ascending limb and early distal convoluted tubule. It is currently recognized as a multifaceted player in kidney physiology and disease, with discrete roles for intracellular, urinary, interstitial and serum uromodulin. Among these, uromodulin modulates renal sodium handling through the regulation of tubular sodium transporters that reabsorb sodium and are targeted by diuretics, such as the loop diuretic–sensitive Na^+^-K^+^-2Cl^−^ cotransporter type 2 (NKCC2) and the thiazide-sensitive Na^+^/Cl^−^ cotransporter (NCC). Given these roles, the contribution of uromodulin to sodium-sensitive hypertension has been proposed. However, recent studies in humans suggest a more complex interaction between dietary sodium intake, uromodulin and blood pressure. This review presents an updated overview of the uromodulin's biology and its various roles, and focuses on the interaction between uromodulin and sodium-sensitive hypertension.

## INTRODUCTION

Uromodulin, also known as Tamm–Horsfall protein, is a kidney-specific glycoprotein which is exclusively produced by the epithelial cells lining the thick ascending limb (TAL) (85%–90%) and early distal convoluted tubule (DCT) (10%–15%), and is released bidirectionally to urine and the interstitial space/circulation [1]. Uromodulin is the most abundant (>50%) urinary protein in healthy individuals, having excretion rates of up to 200 mg/day [2, 3]. It forms polymers in normal urine [4] and is a key component of hyaline casts [5].

Although it was discovered by Tamm and Horsfall in 1950 [6], the role of uromodulin remained unclear for many years [7]. Over the past two decades, uromodulin has been upgraded to a multifaceted player [8], being involved in various physiological and pathological processes [9, 10]. This largely results from recent genetic studies revealing that mutations in the human uromodulin gene (*UMOD*) leading to intracellular accumulation of mutant proteins cause autosomal dominant tubulointerstitial kidney disease (ADTKD) [11], that specific *UMOD* variants are associated with incident chronic kidney disease (CKD) [12–14] and hypertension [15, 16], and that *UMOD*, among multiple genetic loci, shows the strongest association and largest effect on kidney function [17]. Lately, extensive scientific interest has been directed to the association of uromodulin with sodium-sensitive hypertension, as uromodulin acts as a pivotal regulator of sodium homeostasis.

In this review we provide the more recent insights into uromodulin's biology including its multiple roles and functions with a particular focus on its role in the sodium sensitivity of blood pressure (BP). We also summarize the existing evidence originating from experimental and clinical studies that support a link between uromodulin and sodium-sensitive hypertension, and discuss the potential therapeutic consequences of such a link.

## STRUCTURE OF UROMODULIN

**Figure 1:**
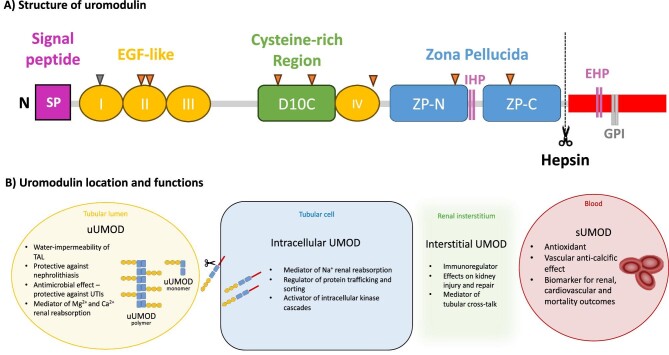
Structure, key domains, and functions of uromodulin. (**A**) Structure and key domains of uromodulin. Note that the eight triangular shapes constitute the N-glycosylation sites; the seven orange triangles represent the occupied glycosylation sites, whereas the sole grey triangle corresponds to the non-used site. (**B**) Key functions of uromodulin.

Uromodulin is synthesized as a 640 amino-acid precursor and has multiple conserved domains [18]. It is consisted by a leader signal peptide (cleaved after targeting nascent uromodulin to endoplasmic reticulum), four N-terminal epidermal growth factor (EGF)–like domains (marked I–IV sequentially), a cysteine-rich region (D10C), a C-terminal bipartite zona pellucida (ZP) (ZP-N and ZP-C), an internal hydrophobic patch within the ZP-N/ZP-C linking region, an external hydrophobic patch (EHP) and a glycosylphosphatidylinositol (GPI)-anchoring site (Fig. 1A). Uromodulin undergoes extensive intracellular post-translational modifications, including N-glycosylation in seven out of eight conserved sites [19], formation of 24 disulfide bridges and cleavage at the C-terminal by the serine protease hepsin [20]. Endoplasmic reticulum plays an important role in uromodulin processing.

Recently, the perplexed structure of uromodulin was further elucidated using cryo-electron microscopy (cryo-EM) [21, 22]. Uromodulin is polymerized into filaments, the core of which is formed by a unique interlocked configuration of ZP-N and ZP-C domains [22], arranged in a helical pattern with ∼65 Å rise and ∼180° twist [21]. ZP-N and ZP-C domains possess an immunoglobulin-like structure and interact with the ZP-linking region through the formation of β-sheets [21]. After hepsin's cleavage and EHP dissociation, uromodulin monomers are incorporated into a growing filament with a head-to-tail orientation, as an activated ZP-C end interacts with the ZP-N domain of an incoming subunit [22]. A subsequent study combining AlphaFold2 predictions with X-ray crystallography and cryo-EM showed that the cysteine-rich region consists of 10 rather than 8 cysteine residues [23].

## AN OVERVIEW OF UROMODULIN ROLES

Uromodulin has pleiotropic roles, regulating numerous molecular and physiological activities, that may be shared across species, as the *UMOD* gene is evolutionary conserved [24].

Importantly, uromodulin is secreted bilaterally through both the apical (tubular lumen) and basolateral (interstitium) membranes. It remains intracellular and/or bound on the apical membrane until cleavage by hepsin releases uromodulin primarily to the tubular lumen, where it forms macromolecular polymers [polymerizing urinary uromodulin (uUMOD)] and less frequently monomers (non-polymerizing uUMOD) (Fig. 1B). A membrane-bound peptide is left behind. A minor fraction of uromodulin is released as monomers in the renal interstitium [interstitial uromodulin (iUMOD)], ultimately reaching the bloodstream [circulating or serum uromodulin (sUMOD)] [1, 25].

uUMOD levels are at least 100- to 300-fold higher than sUMOD levels [26]. Importantly, various genetic loci impact on uUMOD and sUMOD levels by controlling uromodulin transcription, glycosylation, function and clearance. A recent meta-analysis of genome-wide association studies (meta-GWAS) identified that common variants in specific genes, i.e., *UMOD, PDILT, KRT40* and *WDR72*, associate with uUMOD levels [27]. In case of sUMOD, another meta-GWAS using different detection methods (aptamer-based and antibody-based) revealed that *UMOD, PDILT, PRKAG2, B4GALNT2* and genes encoding uromodulin-glycosylating enzymes and/or their receptors determine sUMOD levels [28].

**Figure 2:**
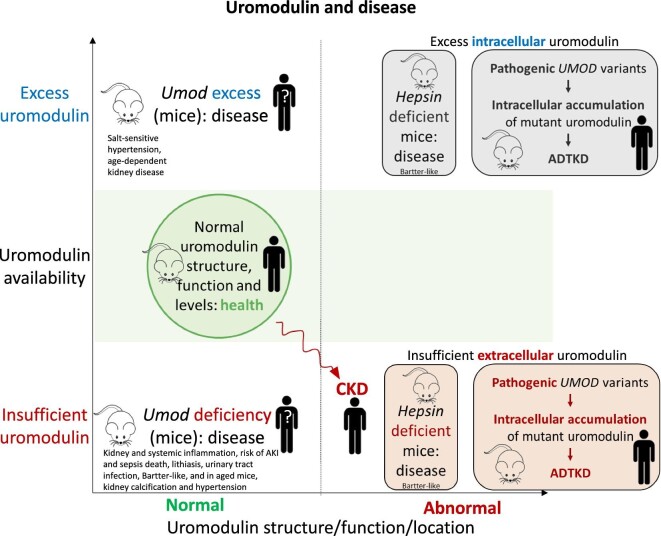
Uromodulin and disease. In CKD, uromodulin production is decreased because of decreased tubular cell mass. Additionally, sUMOD is carbamylated, and this leads to modified functions. As an example, its protective function against vascular calcification is lost. Gain-of-function *UMOD* mutations causing ADTKD typically interfere with uromodulin processing, leading to intracellular accumulation of abnormal uromodulin that causes proteotoxicity, as well as uromodulin deficiency in urine and the circulation [120]. The resultant phenotype of ADTKD is a combination of both decreased production of normal uromodulin and the production of abnormal uromodulin. Mutant uromodulin induces ER stress, unfolded protein response and accelerated apoptosis, leading to tubular cell death, nephron drop-out, fibrosis and progressive CKD. Simultaneously, wild-type uromodulin deficiency results in decreased activation of NKCC2 and annuls the water-impermeability of TAL; therefore, sodium reabsorption is reduced, and hypovolemia occurs. As a compensatory mechanism, it has been hypothesized [121] and in part experimentally demonstrated [122] that sodium reabsorption is increased in the proximal tubule, a process that is coupled with increased proximal urate reabsorption; thus, patients develop hypouricosuric hyperuricemia and gout early in life. Similarly, in hepsin-deficient mice, there is a combination of excess intracellular uromodulin causing cytotoxicity and insufficient extracellular uromodulin, potentially leading to features of uromodulin insufficiency.

The different uromodulin forms play discrete roles, which are discussed below in detail. However, experimental systems do not always allow clear differentiation of the respective roles of intracellular uromodulin, iUMOD, sUMOD and uUMOD. Thus, evidence has been gathered from uromodulin-deficient mice (that are missing all uromodulin forms) [29–33], cultured cells overexpressing uromodulin in the cell membrane among other sites [34], mice that do not secrete uromodulin resulting in cytotoxic intracellular accumulation [35, 36], interstitial cells exposed to uUMOD or uUMOD polymers [37, 38], which may not represent the actual nature or concentration of iUMOD, or even the parenteral administration of truncated uromodulin isoforms from urine (i.e., using a uUMOD source to study the systemic effects of sUMOD) [33]. However, it remains relevant to try to dissect systemic from intracellular or urine actions of uromodulin, as eventual therapeutic intervention may differ. Additionally, the pathogenesis of ADTKD appears mainly related to proteotoxicity resulting from the intracellular accumulation of mutant proteins [9, 39], limiting the information that may be derived on the function of uromodulin in humans. In this regard, *Umod*-deficient (*Umod^−^^/^^−^*) mice do not present with histological changes associated with ADTKD [40], indicating that the resultant phenotype of ADTKD is mainly due to a gain-of-function effect. *Umod^−^^/^^−^* mice do not develop full-blown CKD, but display spontaneous neutrophilic kidney and systemic inflammation and oxidative stress [32, 41], which may interfere with the study of uromodulin function *in vivo*. To complicate matters further, several forces may interfere with the interpretation of clinical studies. On one hand, as TAL mass is lost and CKD progresses, sUMOD and uUMOD decrease [3, 42]. However, in individuals with CKD, sUMOD undergoes further post translational modifications, such as urea-driven carbamylation, which may lead to loss or switch of function [43, 44]. On the other hand, the *UMOD* locus is a key genetic risk factor for renal traits [9]. Thus, risk alleles in the *UMOD* promoter region or other genes are associated with higher uUMOD levels, raising the possibility that life-long excess uromodulin production is detrimental, as supported by transgenic mouse studies discussed below [9, 16], although promoter gene variants may also modulate the expression of (uncharacterized) non-*UMOD* genes [45]. Overall, uromodulin deficiency, excess or abnormality may be disease-causing (Fig. 2), but the precise details and clinical practice translation requires further research.

### Cellular (intracellular and cell-surface) uromodulin

In TAL cells, intracellular uromodulin regulates protein trafficking and sorting, organizes the lipid microdomains of the apical membrane through its GPI-anchoring site [46, 47] and activates intracellular kinase cascades [16]. Through these actions, intracellular uromodulin regulates the tubular handling of electrolytes.

**Figure 3:**
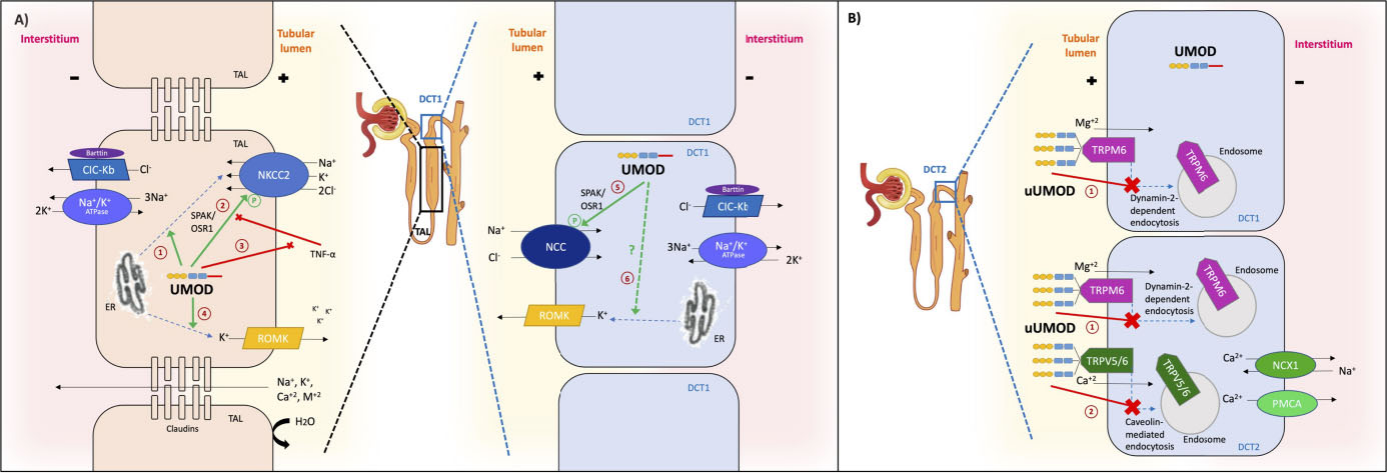
Uromodulin location and interaction with key electrolyte transporters. (**A**) Interaction with sodium transporters. Uromodulin is produced by the epithelial cells lining the TAL and early DCT (DCT1) and remains intracellular until a final cleavage by hepsin releases it in the lumen. In urine, uromodulin mostly forms macromolecular polymers, but can be also found in monomers. In TAL, uromodulin increases Na^+^ reabsorption by activating apical NKCC2 through numerous mechanisms. It mediates vesicular translocation of NKCC2 from the endoplasmic reticulum (ER) to the apical membrane (1), enhances NKCC2 phosphorylation by activating SPAK/OSR1 kinases (2), counteracts the inhibitory effects of TNF-α on NKCC2 (3) and upregulates the expression of ROMK channels by promoting their vesicular translocation from ER to apical membrane (4). In DCT1, uromodulin additionally regulates Na^+^ handling by activating NCC by promoting its phosphorylation through SPAK/OSR1 (5) in addition to likely upregulating ROMK channel expression (6). (**B**) Interaction with key calcium and magnesium transporters. Uromodulin modulates Mg^2+^ and Ca^2+^ reabsorption by DCT, as uUMOD orchestrates the formation of a urinary multi-protein complex (lattice) that reduces the dynamin-2-dependent endocytosis of TRPM6 (1) and caveolin-mediated endocytosis of TRPV5/6 channels (2) in DCT1 and DCT2; thus, the surface abundance of these transporters is increased.

Uromodulin increases distal sodium (Na^+^) reabsorption by activating the furosemide-sensitive, TAL apical Na^+^-K^+^-2Cl^−^ cotransporter type 2 (NKCC2) [48], which is considered to be involved in the sodium sensitivity of BP (Fig. 3A) [49]. NKCC2 inactivation causes the salt-losing Bartter syndrome. NKCC2 may be activated directly through increased surface expression and phosphorylation or indirectly through activation of renal outer medullary K^+^ (ROMK) apical channels [50]. Uromodulin promotes phosphorylation of NKCC2 by SPAK (STE20/SPS1-related proline/alanine rich kinase) and OSR1 (oxidative stress response 1) kinases [16]. This action is facilitated by a chloride-sensing mechanism, as uromodulin induces phosphorylation of NKCC2 under low chloride hypotonic stress [51]. Moreover, uromodulin counteracts the deactivating effects of inflammatory cytokines such as tumour necrosis factor (TNF)-α [43, 52], which inhibits NKCC2 expression and phosphorylation [53–55]. Additionally, uromodulin may mediate the vesicular translocation of NKCC2 from the endoplasmic reticulum (ER) to the apical membrane [56]. Finally, uromodulin increases the apical membrane expression of ROMK, which creates the K^+^ conductance that allows NKCC2 simultaneous reabsorption of K^+^, Na^+^ and 2Cl^−^ [57].

In parallel, uromodulin activates the thiazide-sensitive, apical Na^+^/Cl^−^ cotransporter (NCC) in the early part of DCT, by promoting its SPAK/OSR1-mediated phosphorylation [58]. NCC inactivation causes the salt-losing Gitelman syndrome.

Lastly, cellular uromodulin seems to also facilitate neutrophil migration across renal epithelial monolayers, which may improve the response against urinary tract infection [34].

### Urinary uromodulin (uUMOD)

uUMOD is the most abundant uromodulin form, considered to be responsible for creating and preserving the water-impermeability of TAL through the formation of a hydrophobic electronegative gel-like seal that spreads over the tubular lumen. As a result, it modulates urine concentration and sustains the countercurrent mechanism for free water conservation [59].

Additionally, uUMOD protects against nephrolithiasis and prevents calcium (Ca^2+^) oxalate and phosphate crystals formation by binding Ca^2+^ on its highly negatively charged sialyl residues [60]. In a large Mendelian randomization study, 1-unit higher genetically predicted uUMOD indexed to creatinine was linked to lower risk of kidney stone formation [odds ratio (OR) = 0.62, 95% confidence interval (CI) 0.55–0.7]; of note, this protective role may be partially mediated by glomerular filtration rate (GFR) (β = –0.09, 95% CI −0.13 to −0.06; mediation proportion = 20%) [61].

Moreover, uUMOD 3D polymers trap pathogens, protecting from urinary tract infections [62], as shown in preclinical models [63–65] and confirmed in clinical settings [66, 67]. Besides, uUMOD protects the urothelial permeability barrier by electrostatically neutralizing cations that could injure urothelium [68].

Lastly, uUMOD regulates the renal reabsorption of magnesium (Mg^2+^) and Ca^2+^ in DCT and connecting tubules. It reduces the endocytosis and upregulates the apical expression of epithelial magnesium channel transient receptor potential subfamily M member 6 (TRPM6) [69] and transient receptor potential cation channel subfamily V member 5 and 6 (TRPV5/6) [70], decreasing urinary calcium and magnesium excretion (Fig. 3B).

Loss of uUMOD is thought to represent kidney injury/loss of kidney function and is associated with adverse outcomes. At the time of kidney biopsy, higher uUMOD is independently associated with less severe histologic findings of interstitial fibrosis/tubular atrophy (–2.5%, 95% CI –4.6% to –0.4% per 2-fold difference in uUMOD), irrespective of kidney function [estimated GFR (eGFR)] and damage (albuminuria) markers; therefore, uUMOD can be used as a biomarker for tubulointerstitial kidney fibrosis [71]. In the elderly, higher uUMOD levels are independently associated with lower risk for eGFR decline (OR = 0.77, 95% CI 0.62–0.96) [42] and all-cause mortality [hazard ratio (HR) = 0.90, 95% CI 0.83–0.98] [42], whereas in CKD patients, lower uUMOD is linked with higher risk for incident kidney failure/rapid eGFR decline (HR = 3.589, 95% CI 1.002–12.992; *P* = .011) [72].

### Interstitial uromodulin (iUMOD)

Uromodulin is thought to be a major kidney immunoregulator and contribute to both kidney injury and kidney repair. The precise role of iUMOD may depend on the local microenvironment at each stage of kidney injury and the amount and specific forms of iUMOD, although this has not been well characterized. Upon TAL cell injury, released iUMOD may act as a danger-associated molecular pattern (DAMP) recruiting innate immunity through the phagocytosis of uromodulin nanoparticles by monocytes, leading to NLRP3 (NOD-, LRR- and pyrin domain-containing protein 3) inflammasome-dependent interleukin-1β production [38] or the activation of Toll-like receptor-4 in dendritic cells [37]. Therefore, iUMOD links innate and adaptive immunity [37], resulting in immunostimulatory effects. Uromodulin may interact with other scavenger receptors in macrophages [73] and induce mononuclear phagocyte proliferation and phagocytosis [33, 74]. Of interest, innate immune system abnormalities observed in uromodulin-deficient mice are more marked in the interstitial space between injured S3 proximal segments and TAL, suggesting that iUMOD contributes to tubular cross-talk during kidney physiology and repair [29, 30, 75].

### Circulating or serum uromodulin (sUMOD)

sUMOD is considered a biomarker of tubular integrity and mass [76] and has been proposed as a putative biomarker for renal, cardiovascular and mortality outcomes.

sUMOD decreases in the early stages of tubular atrophy and interstitial fibrosis in patients with glomerulopathies [77]. Among 426 participants (355 patients with CKD G1–G4 and 71 controls), sUMOD was strongly correlated with eGFR (β = 0.696, 95% CI 0.603–0.719) [78]. Additionally, it was the only parameter significantly improving a model of demographic variables to identify patients with CKD G1 [area-under-the-curve (AUC) = 0.831, 95% CI 0.746–0.915], while serum creatinine, urea or cystatin C were not informative [78]. Moreover, higher pre-operative sUMOD levels were associated with a decreased risk of post-operative acute kidney injury (AKI) [79, 80]. Higher sUMOD was independently associated with decreased risk of renal function deterioration and CKD progression to kidney failure in the elderly (OR = 0.75, 95% CI 0.60–0.95) [81], patients with CKD (HR = 0.24, 95% CI 0.10–0.55 in highest vs lowest sUMOD quartile) [82] and patients with coronary artery disease (CAD) (OR = 0.263, 95% CI 0.087–0.799) [83]. In kidney transplant recipients, low pretransplant [84] and posttransplant [85] sUMOD was independently associated with higher odds (OR = 4.41, 95% CI 1.54–13.93 for lowest vs highest sUMOD quartile) and risk for allograft failure (HR = 2.00, 95% CI 1.06–3.77), respectively. Low sUMOD indexed by eGFR was associated to increased odds for renal flare in patients with lupus nephritis (OR = 2.91, 95% CI 1.21–6.98; *P* = .02) [86].

Additionally, sUMOD has been linked to hard endpoints. Higher sUMOD is independently associated with lower risk for cardiovascular events and overall mortality in the elderly (HR = 0.89, 95% CI 0.80–0.99 and HR = 0.80, 95% CI 0.67–0.96, respectively) [87], and patients with CKD (HR = 0.57, 95% CI 0.38–0.87 and HR = 0.63, 95% CI 0.45–0.90, respectively) [82] or CAD (HR = 0.57, 95% CI 0.37–0.89) [88], even after adjustment for eGFR and albuminuria.

sUMOD is also strongly associated with other adverse systemic outcomes. Thus, it is inversely associated with impaired glucose metabolism [89]. In adolescents with type 1 diabetes, decreased sUMOD is linked to higher ascending aortic pulse wave velocity (β = −0.039, 95% CI −0.017 to −0.062; *P* = .007) [90]. In septic patients, higher sUMOD levels correlated with critical illness [74].

sUMOD concentrations were also inversely correlated with serum calcification propensity, and it counteracts medial vascular calcification through binding to inflammatory pro-calcific cytokines [43]. Interestingly, sUMOD did not protect CKD mice against vascular calcification, likely because it was post-translationally modified by carbamylation [43].

*Umod^−^^/^^−^* mice develop normal kidneys but display urinary salt wasting, low GFR, and spontaneous kidney and systemic oxidative stress and inflammation, mainly characterized by neutrophilia, infiltration of kidney and other organs by neutrophils and increased kidney expression and/or urinary excretion of multiple chemokines and cytokines [32, 41, 91, 92]. Despite evidence of spontaneous inflammation, the number of kidney macrophages was lower than in wild-type (WT) mice [33]. The neutrophilic kidney inflammation was associated with increased sensitivity to AKI induced by ischaemia–reperfusion injury and an underrepresentation of M2 repair macrophages and also increased sepsis bacterial burden and mortality [29, 31, 33, 74]. The parenteral administration of a truncated form of human uUMOD improved AKI, macrophage phenotype and antibacterial properties and decreased sepsis mortality, suggesting a kidney and antibacterial protective role of sUMOD (or more precisely, ‘circulating uUMOD’) [33, 74]. In this regard, upon induction of AKI, sUMOD decreases [41], and during recovery from AKI, uromodulin is redirected from the apical towards the basolateral membrane and is associated with increased sUMOD, but not uUMOD levels [31]. The systemic oxidative stress in *Umod* knock-out (KO) mice is thought to result from sUMOD inhibition of TRPM2, a nonvoltage-activated, Ca^2+^-permeable, nonselective cation channel that plays a role in oxidative stress-coupled diseases [41]. Over time, *Umod* KO mice develop kidney calcification [56] and may no longer demonstrate uromodulin function, but the consequences of kidney disease.

## UROMODULIN AND SODIUM-SENSITIVE HYPERTENSION

Based on the Guytonian model of pressure-natriuresis, high sodium intake leads to increased BP that maintains homeostasis through urinary sodium excretion (natriuresis) [93]. Although in the majority of normotensive and hypertensive patients the required increase in BP is small, in some cases pressure-natriuresis is impaired and an increase in dietary sodium triggers disproportionate increases in BP, the so-called sodium sensitivity of BP [94]. Impaired renal circulation (renal vasoconstriction due to reduced endogenous nitric oxide [95] and renal kallikrein [96]), blunt suppression of renin–angiotensin–aldosterone system [97], sympathetic nervous system overactivity [98], paradoxically reduced levels of atrial natriuretic peptide [99] and hyperinsulinemia [100] represent the main pathophysiologic mechanisms.

Accumulated evidence has suggested that uromodulin plays a central role in the development of sodium-sensitive hypertension. The physiologic substrate lies in the tight functional connection of uromodulin with Na^+^ handling through modulation of NKCC2 and NCC transporters’ activity. In the following lines, we present the main preclinical, genetic and clinical studies linking uromodulin to hypertension and sodium sensitivity.

### Preclinical studies

Preclinical studies were mainly performed in animals with uromodulin deficiency or overexpression. *Umod^−^^/^^−^* mice (uromodulin deficiency) display a urine concentrating defect, polyuria, salt wasting, low BP, low eGFR and a compensatory upregulation of distal tubular sodium transporters (some misplaced in the cytoplasm, like NKCC2 and NCC, rather than in the apical membrane) [91, 92]. Low GFR likely depends on tubuloglomerular feedback due to high amounts of salt at the macula densa; consequently, GFR increased upon salt loading [92]. Aging results in kidney calcification, hypertension and oliguria, which was unresponsive to furosemide [101]. However, these findings are difficult to interpret given the anatomical defect and the lack of information on kidney function [56, 101]. In young mice, defective cell membrane NKCC2 likely contributed to polyuria and salt wasting, since furosemide only elicited a partial diuretic natriuretic response as compared with the full response observed in WT mice [56].

**Figure 4:**
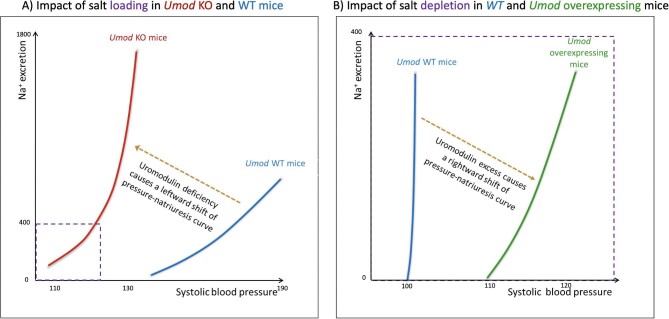
Uromodulin and the pressure-natriuresis curve. (**A**) Uromodulin deficiency causes a leftward shift of pressure-natriuresis curve compared with wild-type (WT) mice when mice are salt-overloaded (2% NaCl in drinking water), indicating that uromodulin deficiency results in the loss of the sodium-sensitivity of blood pressure observed in normal (WT) mice: in the presence of uromodulin, higher sodium intake results in higher blood pressure values than in absence of uromodulin (modified from Graham *et al.* [92]). (**B**) Uromodulin overexpression causes a rightward shift of pressure-natriuresis curve compared with WT mice when mice are salt-depleted (20-fold decrease in dietary NaCl), indicating that uromodulin excess results in the acquisition of the sodium-sensitivity of blood pressure. On the contrary, in normotensive WT mice no effect on BP is evident under these experimental conditions (figure drawn with data from Trudu *et al*. [16]). Na^+^ excretion in µmol/24 h and systolic blood pressure (SBP) in mmHg. Inset in (A) displays the dimensions of panel (B). Notice that studies in panels (A) and (B) used different mouse strains and protocols.

In young *Umod^−^^/^^−^* mice, the low baseline BP did not increase upon chronic 2% NaCl in drinking water (comparable to sea water), differing from the 33% higher BP observed in WT mice [92]. The failure to increase BP was attributed to the high urinary excretion of Na^+^, K^+^ and Cl^−^ (to levels 2- to 3-fold higher than in WT mice) in response to salt loading, resulting in a leftward shift of pressure-natriuresis curves; lower BP values were already associated with higher urinary sodium excretion (Fig. 4A) [92]. Thus, *Umod^−^^/^^−^* mice displayed a Bartter-like phenotype and BP was insensitive to sodium load.

Hepsin-deficient mice present with defective uromodulin processing, as they cannot cleave uromodulin, leading to intracellular uromodulin accumulation, ER stress and tubular damage, as well as low uUMOD [36]. To some extent, they recapitulate cellular events in ADTKD and the phenotype results from both tubular cell injury and low uUMOD. Salt loading (2% NaCl in drinking water) further increased intracellular uromodulin accumulation and cytotoxicity, causing evident histological tubular injury and a Bartter-like syndrome characterized by urinary salt-wasting and stable BP, while WT mice displayed salt-sensitive hypertension, reproducing results from *Umod^−^^/^^−^* mice [92].

The findings are quite different in animal models overexpressing the *Umod* gene. In animals overexpressing the *Umod* gene, there are transgenic mice overexpressing one (heterozygous) or two (homozygous) genes. In this model, there is a dose-dependent higher NKCC2 activity than that in control mice, leading to increased tubular Na^+^ reabsorption and hypertension, left ventricular hypertrophy and histological CKD with apparently preserved kidney function [16]. Furosemide inhibition of NKCC2 increased natriuresis and lowered BP levels more in hypertensive *Umod* transgenic than in normotensive control mice [16]. In *Umod* transgenic mice, hypertension was *Umod* dose-dependent and salt-sensitive, as BP was normalized after an extremely low (>20-fold decrease) salt-restricted diet leading to undetectable urinary Na^+^, while BP did not change upon a low salt diet in WT mice (Fig. 4B) [16].

As summarized above, salt loading triggered uromodulin accumulation in TAL cells from hepsin-deficient mice [36]. These results are in line with two studies in rats, in which salt loading increased uromodulin kidney mRNA and medullary protein in normotensive Sprague–Dawley rats [102] and intracellular uromodulin retention in hypertensive [stroke-prone spontaneously hypertensive rat (SHRSP)] and normotensive rats (Wistar–Kyoto) [103]. In the latter study, 24-h uUMOD excretion was studied and decreased upon salt loading in both rat strains [103]. However, some discrepancies were noted as in the latter study, total kidney (as opposed to medullary) uromodulin protein was unchanged by sodium loading and mRNA was unchanged or even decreased. On the other hand, administration of furosemide—but not chlorothiazide—in normotensive rats on high salt diet resulted in larger increments in kidney uromodulin mRNA levels than high salt diet alone [102].

Overall, studies in genetically modified mice explored extremes in uromodulin availability (from none to 2.5-fold higher than control) and Na^+^ intake (from undetectable urinary Na^+^ to drinking the equivalent of sea water), and lacked a common definition of sodium sensitivity of BP, since WT mice exhibited different BP responses to salt, depending on the experimental setup, rendering the clinical translation difficult [16, 92]. However, the overall message is that lack of uromodulin causes decreased BP that is resistant to dietary salt due to a Bartter-like, urinary salt-wasting phenotype, while unregulated uromodulin excess causes hypertension due to increased tubular Na^+^ reabsorption that is responsive to furosemide. It is unclear how this translates to a physiological environment, where normal uromodulin production is regulated. However, incomplete preclinical evidence also suggested that a high dietary sodium intake may increase uromodulin, setting the scenario for unwanted tubular Na^+^ retention.

### Genetic human studies

Genetic human studies of the past two decades have proposed a strong relationship of *UMOD* gene variants with sodium sensitivity and hypertension. Specific *UMOD* variants are associated with decreased or increased risk for hypertension (see Table 1), as confirmed in large genetic Mendelian randomization studies.

**Table 1:** Genetic studies linking *UMOD* gene variants with hypertension.

*UMOD* variant	Variant location	Allele	Effect on uUMOD levels	Author, year	Participants	Main results
rs13333226A>G	5′ region, promoter	A: risk G: protective	↑ ↓	Padmanabhan *et al*., 2010 [15]	21 466 European hypertensive cases vs 18 240 controls• Discovery cohort: 1621 cases vs 1699 controls• 2 validation cohorts: 19 845 cases vs 16 541 controls	For G allele:• Unadjusted OR = 0.87 (95% CI 0.84–0.91) for hypertension• Adjusted (for age, sex, BMI) OR = 0.85 (95% CI 0.81–0.89) for hypertension• Adjusted (for age, sex, BMI, eGFR) OR = 0.893 (95% CI 0.83–0.96) for hypertension• ↓ 0.49 mmHg SBP (*P* = 2.6 × 10^−5^) and ↓ 0.30 mmHg DBP (*P* = 1.5 × 10^−5^) for each copy of allele
		A: protective G: risk	$\leftrightarrow $	Han *et al*., 2012 [104]	910 Chinese, general population	For G allele:• $\leftrightarrow \ $SBP (mean ± SD: A/G + G/G: 139.6 ± 18.1 vs A/A: 138.1 ± 17.6 mmHg; *P* = .356)• ↑ DBP (A/G + G/G vs A/A: 83.1 ± 10.7 vs 81.2 ± 10.2; *P* = .046)
rs6497476T>C	5′ region, promoter	T: protectiveC: risk	$\leftrightarrow $	Han *et al*., 2012 [104]	879 Chinese, general population	For C allele:• ↑ DBP (mean ± SE_m_: C/C: 96.0 ± 3.9 mmHg vs T/C: 86.5 ± 1.2 mmHg vs T/T: 84.8 ± 0.4 mmHg; *P* = .025)
rs4293393T>C	5′ region, promoter	T: risk	↑	Trudu *et al*., 2013 [16]	471 untreated hypertensives (mixed ethnic groups)	For T allele:• Baseline BP (T0min): $\leftrightarrow {\mathrm{\ }}$SBP (mean ± SD: C/C + C/T: 141 ± 0.80 vs T/T: 142 ± 0.63 mmHg; *P* = ns), ↑ DBP (C/C + C/T: 91 ± 0.66 vs T/T: 93 ± 0.50; *P* = .011)• Furosemide response (T240 min): $\leftrightarrow {\mathrm{\ }}$SBP/DBP (C/C + C/T: 145 ± 2.40/99 ± 1.57 vs 144 ± 1.58/99 ± 0.92; *P* = ns for both), ↑ natriuresis (C/C + C/T: 565 ± 140 vs T/T: 629 ± 177 μmol/min; *P* = .024)• Delta (T240–T0): marginally ↑ ΔSBP (C/C + C/T: –0.9 ± 1.61 vs T/T: –4.3 ± 0.95; *P* = .06), ↑ ΔDBP (C/C + C/T: 0.47 ± 1.00 vs T/T: –2.0 ± 0.64; *P* = .037)
		C: protective	↓			
rs4293393A>G	5′ region, promoter	A: riskG: protective	NA	Akwo *et al*., 2022 [105]	648 593 veterans	For the A allele:• OR = 1.03 (95% CI 1.02–1.05) for hypertension
rs12917707G>T, A	5′ region, promoter	G: risk T: protective	↑ ↓	Prudente *et al*., 2017 [106]	3087 type 2 diabetes (4 Italian cohorts)	For T allele:• Unadjusted OR = 0.86 (95% CI 0.75–0.99) for hypertension• Adjusted (for eGFR) OR = 0.87 (0.75–1.01) for hypertension
		G: protectiveA: risk	NA	Wang *et al*., 2021 [107]	514 Chinese (Baoji Salt-Sensitive Study cohort)	For rs12917707:• Significant association with longitudinal SBP changes on recessive model (over 4 years: β=0.009, over 8 years: β = 0.023; *P* < .05 for both)
rs7193058G>A	Exonic	G: protectiveA: risk	NA	Du *et al*., 2021 [108]	80 Chinese adults (16 hypertensive vs 64 normotensive)	For rs7193058:• Significant association with DBP response on high-salt diet (β = 0.223; *P* = .042)
rs4997081C>G	Intronic	C: riskG: protective	NA	Du *et al*., 2021 [108]	80 Chinese adults (16 hypertensive vs 64 normotensive)	For rs4997081:• Significant association with DBP response on high-salt diet (β = −0.311; *P* = .015)• Significant effect from eGFR on BP
rs12708631T>A	Intronic	T: protectiveA: risk	NA	Wang *et al*., 2021 [107]	514 Chinese (Baoji Salt-Sensitive Study cohort)	For rs12708631:• Significant association with longitudinal changes in SBP (over 4 years: β = 0.012, over 8 years: β = 0.030; *P* < .05 for both) and DBP (over 4 years: β = 0.006, over 8 years: β=0.020; *P* < .05 for both) on recessive model

BMI, body mass index; NA, not available/not reported; ns, not significant; SD, standard deviation.

In a GWAS including a large sample of 21 466 hypertensive cases and 18 240 controls [15], a minor G allele in the *UMOD* gene promoter rs13333226 was associated with lower uUMOD, lower BP levels and lower risk for hypertension incidence [15]. A smaller (*n* = 910) cross-sectional Chinese study in the general population did not examine the association with hypertension risk, but showed slightly higher diastolic BP (DBP) for the G allele [104]. These findings are inconsistent with the fact that minor G allele codes for lower uUMOD and consequently lower BP levels would be expected. These contradictory findings may be related to different populations under study; the first study included a sample from general population, whereas the second one included hypertensive patients.

Moreover, in almost 650 000 veterans, the major T (or A) was the risk allele for higher hypertension incidence in *UMOD* promoter rs4293393, compared with the G (or C) allele [105]. In 471 untreated hypertensive patients, furosemide led to higher natriuresis and larger decrease in BP in patients homozygous for the T risk allele than in carriers of the C protective allele [16]. Finally, regarding rs12917707 *UMOD* promoter variant, the minor T allele was associated with lower hypertension risk in type 2 diabetic patients compared with G allele [106]; this variant was significantly associated with longitudinal systolic BP (SBP) changes over an 8-year follow-up in a large Chinese cohort [107]. A relationship with BP levels was also reported for rs6497476 [104], rs7193058 [108], rs4997081[108] and rs12708631 [107] (see Table 1). rs7193058 is an exonic *UMOD* variant, rs4997081 and rs12708631 are intronic *UMOD* variants, while rs6497476 is a variant located in the *UMOD* promoter.

In addition to the above, solid evidence has been recently gained from large Mendelian randomization genetic studies. A two-sample Mendelian randomization study on four GWAS consortia including approximately 750 000 Europeans investigated the causal association between BP and uUMOD levels, as predicted by the *UMOD* promoter variant rs12917707 and the *PDILT* intronic variant rs4494548 (located upstream of *UMOD*) [109]. Higher predicted uUMOD levels were significantly associated with lower eGFR, higher odds for eGFR decline or CKD, and higher SBP or DBP. Single nucleotide polymorphisms (SNPs) associated with each 1-SD higher uUMOD were associated with an increase in SBP by 0.06 and DBP by 0.082 SD, but no reverse causal effect was detected. Of note, the effect of uUMOD on higher BP was mediated by lower eGFR, suggesting that it was not a direct consequence of uUMOD itself, but of CKD [109].

A study with over 1 000 000 participants revealed that hypertension incidence is linked to the same SNPs as in the prior study associated with high uUMOD and to 16 SNPs associated with high sUMOD levels (OR = 1.013, 95% CI 1.009–1.0018; *P* < .001) [110]. In this analysis, higher predicted uUMOD and sUMOD levels were causally associated with higher SBP and DBP, but it did not report on the influence of eGFR [110].

Finally, a third study assessing the relationship between the synonymous *UMOD* rs13335818 and the *PDILT* rs77924615 gene variants associated to uUMOD and cardiovascular events showed that a higher predicted uUMOD/urinary creatinine (uCr) ratio was associated with increased SBP/DBP but no reverse relation was shown [111]. Importantly, in mediation analysis, the effect of uUMOD on myocardial infarction was mainly mediated by SBP and DBP [111].

In conclusion, genetic human studies have identified robust associations of *UMOD* with sodium sensitivity and hypertension, as specific *UMOD* variants increase uUMOD and BP levels and the risk for incident hypertension. Comprehensive Mendelian randomization studies, involving large and diverse cohorts, further affirm these genetic associations by highlighting the causal relationship between *UMOD* variants, uUMOD levels and hypertension. As the impact of uUMOD on BP appears to be mediated by eGFR levels, the predisposition to hypertension harboured by specific *UMOD* variants may be potentially modulated by CKD.

### Clinical studies

Two studies have already investigated the association of uUMOD and BP response to salt intake in the general population. In an interventional study in 30 healthy individuals who were genetically predisposed to hypertension, 1 week of low-salt (10 mmol sodium/day) was followed by 1 week of high-salt (240 mmol/day) diet [112]. The 12-h nighttime uUMOD excretion rate during low-salt was lower than baseline or during high-salt diet, but 24-h uUMOD excretion rates did not differ, meaning that daytime uUMOD excretion differences occurred in the opposite direction than nighttime ones [112]. After high-salt diet, subjects with sodium sensitivity of BP above the median presented higher 24-h uUMOD than those with sodium sensitivity below the median, and sodium sensitivity correlated moderately with uUMOD/uCr ratio (r = 0.37, *P* < .05), but apparently not to 24-h uUMOD which is a more relevant variable [112].

The association of uUMOD with sodium sensitivity was further supported by a recent study in 948 European adults [113]. Individuals with 24-h uUMOD above the sex-specific median presented a significant adjusted association of higher 24-h urinary Na^+^ excretion with 24-h SBP and a non-significant trend of higher DBP levels [113]. By contrast, participants with uUMOD below the median exhibited a significant association of higher 24-h urinary Na^+^ excretion with lower 24-h ambulatory DBP levels [113]. These results were the first to show the association of uUMOD with either sodium sensitivity or inverse sodium sensitivity, depending on uUMOD levels. The association of 24-h uUMOD levels with both *UMOD* rs12917707 variants (i.e., G and T alleles) was confirmed and subjects with higher uUMOD had higher eGFR [113]. Interestingly, not only Na^+^ excretion, but also higher urine volume (e.g., following water loading) increased 24-h uUMOD excretion without changing uUMOD concentration [114].

The data supporting a link between uromodulin and sodium sensitivity in hypertensive patients are more extensive. A preliminary interventional study showed no difference in baseline 24-h uUMOD levels between 65 hypertensive and 23 normotensive patients [115]. However, in hypertensive patients, uUMOD increased after furosemide (*n* = 24) for 10 days but not after nifedipine (*n* = 21) or propranolol (*n* = 20) [115]. In newly diagnosed and untreated hypertensive males (19 sodium-sensitive, i.e., SBP increased >4 mmHg after an acute 2 L saline infusion, 37 sodium-resistant), uUMOD levels assessed by western blot in spot urine samples were higher in hypertensive patients than in healthy controls (both *P* < .001); however, there were no differences between hypertensive patient groups [116]. uUMOD predicted hypertension with an AUC for the receiver operating characteristic curve of 0.793 (95% CI 0.679–0.879) to 0.804 (95% CI 0.696–0.904) for both hypertension groups. Urinary samples with lower uUMOD levels had higher urinary Na^+^ concentration [116]. Unfortunately, the methods (western blot in spot urine samples) are suboptimal.

Another interventional study [108] determined 24-h uUMOD and sUMOD levels in 16 hypertensive and 64 normotensive Chinese adults that followed consecutively normal, low-salt (50 mmol Na^+^/day, actual mean urinary excretion 91 mmol/day) and high-salt (300 mmol Na^+^/day, urinary excretion 266 mmol/day) diets for 1 week each and overall behaved as sodium-sensitive BP. uUMOD and sUMOD levels were significantly lower during high-salt diet compared with baseline. uUMOD inversely correlated with 24-h urinary Na^+^ excretion while sUMOD did not [108]. However, this study has several major issues, ranging from the ∼50% decrease in uUMOD in low-salt diet versus baseline, which further decreases on high-salt diet, to the extremely low 24-h uUMOD excretion (mean value <3 mg/24 h in the three conditions).

Bakhoum *et al*. [117], in their *ad hoc* analysis of 157 participants in the Dietary Approaches to Stop Hypertension (DASH)-Sodium Trial, did not observe associations between baseline 24-h uUMOD and change in office BP levels in response to three diets of different salt intake (50, 100, 150 mmol Na^+^/day). There was no interaction between baseline uUMOD and dietary Na^+^ contents on end-of-intervention SBP. Furthermore, baseline uUMOD was not associated with SBP change from low- to high-Na^+^ diet [117]. However, the range of dietary sodium was narrow and uUMOD was only assessed at baseline, while dietary sodium may influence uUMOD levels.

Supporting evidence can be extracted from clinical studies in hypertensive patients assessing the response of BP to loop diuretics based on *UMOD* genotype. Following results in transgenic mice [16], Trudu *et al*. retrospectively analyzed a cohort of 471 treatment-naive hypertensive patients stratified *a posteriori* for a *UMOD* risk variant (rs4293393T>C). For a subset of these patients (*n* = 165), data from furosemide tests were available; furosemide administration in patients homozygous for the risk allele (TT) led to greater natriuretic response (i.e., higher natriuresis over baseline and greater BP reduction) as compared with other hypertensive patients (CC + CT) [16]. Thus, the hypothesis that patients with risk *UMOD* alleles will respond better to loop diuretics was proposed. In this regard, an ongoing prospective genotype-directed clinical trial (BHF UMOD Trial) examining whether the hypertensive patients show differential BP response to torasemide based on their *UMOD* genotype is currently testing the above hypothesis [118].

Finally, there is only a paediatric study assessing the relation between hypertension and uUMOD in the setting of CKD [119]. In 436 children (age 6 months to 16 years, eGFR = 30–90 mL/min/1.73 m^2^) no association between uUMOD/uCr and either 24-h or office SBP/DBP were evident in multivariable models and the relationship between uUMOD and BP levels was not modified by adding estimated sodium intake in multivariable models [119].

In summary, clinical studies in both healthy individuals and hypertensive patients indicate a potential link between uromodulin and sodium sensitivity, directly affecting the mean BP levels and BP response to salt intake. In hypertensive patients, uUMOD levels may act both as a prognostic marker and therapeutic target; selected *UMOD* variants could guide the personalized administration of loop diuretics to patients who will benefit the most and the results of ongoing trials testing this hypothesis are awaited to draw firm conclusions. With regards to CKD population, solid data on the link of uromodulin with sodium sensitivity are currently missing from the literature. Methodological variations and inconsistent findings of existing studies underline the need for further research.

## CONCLUSION

Uromodulin facilitates many physiological (renal and systemic) processes, is a useful biomarker predicting clinical outcomes and, most importantly, there is evidence suggesting a role in the sodium sensitivity of BP. These roles, combined with the fact that *UMOD* gene shows the largest effect on renal function, suggest that uromodulin is an irreplaceable player in kidney health and disease. Interventional studies assessing the impact of uromodulin levels on the antihypertensive effect of NKCC2/NCC blockade and/or salt restriction, as well as large population studies investigating the value of adding *UMOD* genotyping in diagnostic and/or therapeutic algorithms of hypertension, are needed. However, given the heterogeneous nature of preclinical and clinical studies so far, several issues remain to be addressed (Box 1) for the success of research efforts.

Box 1. Research needs in the field of uromodulin and sodium-sensitive hypertension.

- In clinical settings, different protocols have been used to identify sodium-sensitive hypertension and up to now, there is not a common, single definition. For these reasons, a consensus with prespecified definition of sodium-sensitive hypertension is needed [94]. It is unclear whether this definition may be replaced in practice by the concept of sodium sensitivity of blood pressure (and not sodium-sensitive hypertension) since a therapeutic intervention will only be needed when hypertension is present.
- The optimal methods to measure uromodulin should be defined and validated, including whether sUMOD or uUMOD should be assessed and for uUMOD, whether in spot, overnight or 24-h samples or normalized for urinary creatinine, sodium or volume.
  - Additionally, it should be determined whether sUMOD or uUMOD should be routinely normalized by a measure of kidney mass or function, in a similar manner to the concept of single-nephron GFR.
  - Eventually, this should crystalize in the definition of an optimal range for sUMOD and uUMOD.
- Assessment of gene variants associated to uromodulin levels could moderate the complexities of measuring uromodulin levels; however, multiple non-genetic factors impacting on uromodulin levels (e.g., age, diabetes, low eGFR, salt intake) should also be considered, as they perplex the applicability and clinical translation of *UMOD* genotyping. Given the multiple associations already described and the fact that risk *UMOD* variants have high allelic frequency in the general population, a consensus should be ideally reached in the future on which gene variants to assess, likely allowing for differences for each genetically distinct population. Whether genotyping of *UMOD* variants can be clinically relevant and informative rests to be investigated by future studies.
- In addition, intracellular uromodulin is not assessed in humans, it may be regulated in opposite direction to extracellular uromodulin and may also influence sodium transporters or promote cytotoxicity. Thus, it remains a known unknown that may be contributing to clinical observations.
- These issues should be agreed in a consensus document by researchers specialized in the field.

## Data Availability

This is a review paper with no new data generated or analysed in support of it.
